# 50 Years since Kaufman and Phillips’ Groundbreaking Trilogy Elucidating Ion and Water Homeostasis in Ixodid Ticks

**DOI:** 10.3390/pathogens12030385

**Published:** 2023-02-28

**Authors:** Ladislav Šimo

**Affiliations:** Laboratoire de Santé Animale, UMR BIPAR, Ecole Nationale Vétérinaire d’Alfort, INRAE, ANSES, F-94700 Maisons-Alfort, France; ladislav.simo@vet-alfort.fr

**Keywords:** salivary glands, physiology, ion, water balance, ticks

## Abstract

The enormous volume of blood ingested by hard ticks during their long attachment period is without a doubt the hallmark of their biology. Maintaining a homeostatic balance between ion and water intake and loss during their feeding is critical to preventing osmotic stress and death. Exactly 50 years ago, Kaufman and Phillips published a series of three consecutive papers on “Ion and water balance in the ixodid tick *Dermacentor andersoni*”, *Journal of Experimental Biology* (1973): I. Routes of ion and water excretion, 58: 523–36; II. Mechanism and control of salivary secretion 58: 537–547; and III. Influence of monovalent ions and osmotic pressure on salivary secretion 58: 549–564. This classic series significantly expanded our knowledge of the unique regulatory processes governing ion and water balance in fed ixodid ticks, highlighting its uniqueness among the blood-feeding arthropods. Their pioneer work had an enormous impact on understanding the vital role of salivary glands in these actions, and ultimately provided a consequential stepping stone for a new era of hard tick salivary gland physiological research.

## 1. Introduction

The hematophagous nature of various arthropod groups necessitates the careful management of overhydration by precise water regulation. The osmoregulatory activity in insects is principally governed via the excretory activities of Malpighian tubules. Although these key mechanisms were established in the early 1930s [1,2,3], the field of insect osmoregulation remains an exciting and challenging research area and is still extensively studied by numerous groups worldwide [4,5,6]. Despite a common homeostatic goal, the same regulatory mechanisms cannot be applied to all blood-feeding arthropod lineages. Specifically, blood-feeding insects engorge relatively quickly, then undergo diuresis and bloodmeal digestion. In their immature stages, this triggers growth, followed by a molt, while in adults, it prompts egg development, then a return to a pre-feeding state [4,5]. In contrast, feeding strategies and excessive water elimination mechanisms in ticks differ to those described in insects. In the case of argasid (soft) ticks, both adult and immature stages feed similarly as blood-sucking insects, in short periods, however, excess water is primarily eliminated by specialized coxal glands that are unique to this tick family [7,8,9]. Furthermore, ixodid (hard) ticks have a distinct bloodmeal uptake strategy that differs from any other hematophagous arthropods. To ensure their biological success, hard ticks feed slowly over a period of days or even weeks, depending on the species and life stage [10,11]. Most hard tick species use a cement cone to secure their mouthparts to the feeding lesion and can consume an enormous volume of blood, resulting in a remarkable increase in body size accompanied by massive fresh cuticle synthesis [12,13]. Repleted fertilized females die after egg laying, while engorged larvae and nymphs molt into nymphs and adults, respectively [10].

Researcher investigating ixodid tick feeding biology in the 1940s speculated that significant amounts of excess fluid could be eliminated by evaporation via the integument, although the author himself did not strongly support this hypothesis [14]. The first suggestion that ixodid tick salivary glands were involved in water regulation was based on the observation of fluid movement in the vicinity of tick mouthparts while attached to live hamster pouch tissue in 1967 [15]. That same year, Tatchell [16] disproved that integument evaporation plays a significant role in water elimination by demonstrating that tritiated water injected into feeding females could be found in the host blood and urine, thus suggesting that the tracer enters the host exclusively via salivary gland secretory activities. The conjecture that salivary glands of hard ticks could play an osmoregulatory role was also proposed based on their progressive increase in size during feeding [17]. Although these reports provided supportive evidence that the integument and salivary glands are involved in osmoregulation, before the 1973 body of work of Kaufman and Phillips [18,19,20] it was not clear (i) whether ticks possess other sites of water loss; (ii) whether salivary glands act in a secretory or filtration-resorptive manner; (iii) which mechanisms control this tissue; and (iv) whether ionic and osmotic factors influence salivary gland secretory activities. The three consecutive publications by Kaufman and Phillips in 1973 [18,19,20], utilizing a series of elegant experiments, shed a completely new light on tick osmoregulation mechanisms, resulting in a breakthrough understanding of the primary importance of salivary glands in these processes. Herein, my aim is to summarize and reiterate their key discoveries and hypotheses in a simple commentary and highlight their powerful impact on tick salivary gland research.

## 2. Discoveries

The classic 1973 trilogy by Kaufman and Phillips [18,19,20] employed a variety of highly specialized approaches to investigate the circulation and excretion of ions and water during the feeding of *D. andersoni* female ticks.

**Figure 1:**
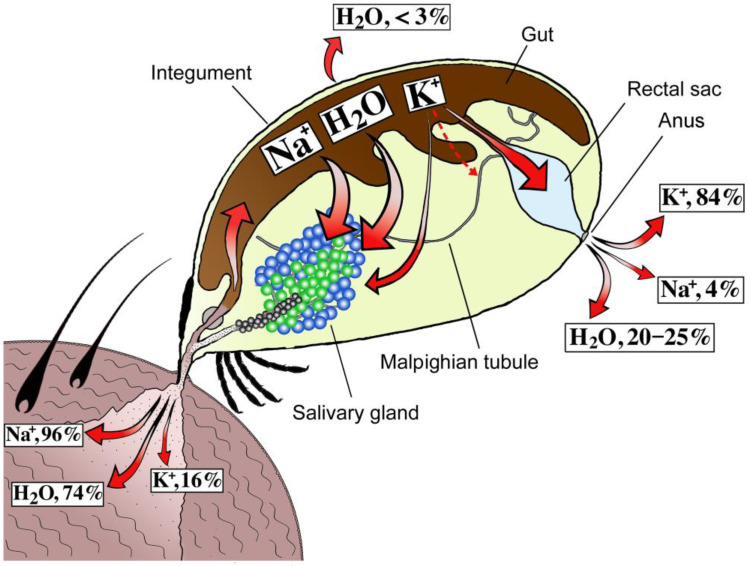
Schematic illustration depicting the injection and elimination of ions and water during *D. andersoni* female feeding. Schema were reconstructed based on Kaufman and Phillips 1973 [18].

The first of the three reports [18] identified salivary glands and the anus as major ion excretion routes (Figure 1). Specifically, the authors clearly demonstrated that 96% of the sodium, 16% of the potassium, and a large proportion of the chloride (no value provided) obtained from a bloodmeal are transported via the gut diverticula to the hemolymph. These electrolytes are subsequently taken up by salivary glands to be secreted into the host in the pool of saliva, while the remainder are largely lost via the anus (Figure 1). The considerable amount of potassium in the feces is likely due to the relative impermeability of the gut wall to potassium ions, an observation that has also been suggested in soft tick studies [21]. This evidence is consistent with the fact that higher concentrations of sodium and chloride have been found in the osmotically stabilized hemolymph in *Dermacentor* females 3–4 days post feeding than in the bloodmeal, while the inverse was observed for potassium. The authors also speculated that Malpighian tubules could play a role in the rapid uptake of potassium from the midgut/hemolymph [18], as this common mechanism has been well-documented in insects [2,5]. It has been suggested that these structures are mainly active in the detachment stage, however, the physiology of tick Malpighian tubules is poorly understood, as the current knowledge is largely based on a single study over 60 years old [22]. Thus, more research is required to fully understand the role of Malpighian tubules in ticks.

Another interesting result arose when the researchers monitored the water balance during *D. andersoni* female tick feeding [18]. Specifically, considerable evidence has shown that approximately 80% of the bloodmeal imbibed by feeding *D. andersoni* females is excreted. Salivary glands are responsible for ~75% of the water lost via saliva injection back into the host, while ~20% is lost via the anus, and only ~3% is evaporated via the integument. Interestingly, in unfed females, the hemolymph demonstrates high ionic content, which dropped to nearly half on the third day of feeding. During engorgement, *D. andersoni* females increased their weight 75 times when compared to their unfed weight, however, the hemolymph volume—that also increases during feeding—always remained stable at 23% of body weight. Authors have hypothesized that this phenomenon is likely to be controlled by regulatory mechanisms, and tested whether salivation itself could act as a regulator. However, directly injecting iso-osmotic saline to force a 25% or 50% increase in hemolymph volume inhibited the spontaneous salivation occurring in non-injected ticks. Thus, the hemolymph volume regulation mechanisms remain obscure.

**Figure 2:**
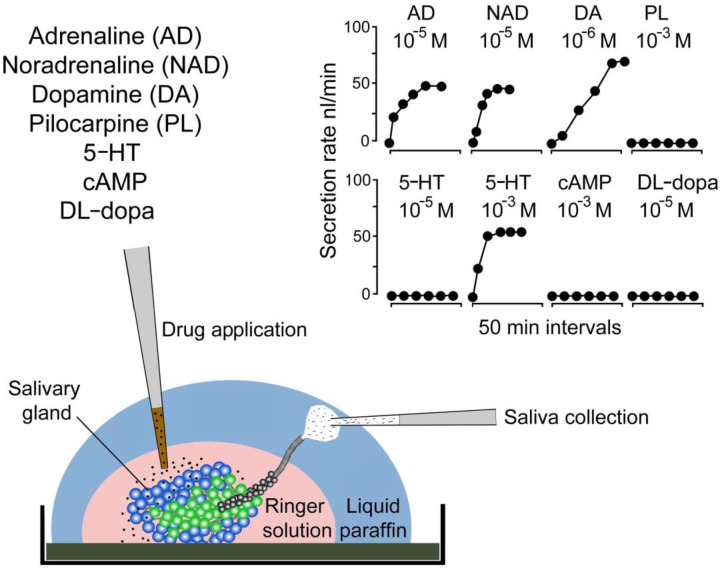
Effect of different drugs on the secretory rate of isolated salivary glands from partially fed *D. andersoni* females. Image summarizes the methodology and shows simplified results described in Kaufman and Phillips 1973 [19].

The second report [19] focused exclusively on the mechanisms controlling salivary gland fluid secretion in *D. andersoni* females (Figure 2). Prior to this study, some groups had already demonstrated that in vivo injection of ticks with the exogenous cholinomimetic drug, pilocarpine, provoked salivation [16,23], however, no information on the response of isolated glands was available. The main reasoning for utilizing an in vitro experiment was to determine whether hard tick salivary glands produce fluids without any external hydrostatic pressure, and thus act as a secretory rather than as a filtration-resorption tissue. In addition, selective testing of different pharmacological agents on salivary secretion was expected to clarify whether salivary glands are under hormonal or neuronal command(s) [19]. Among the multiple agents tested, catecholamines such as dopamine, epinephrine (adrenaline), and norepinephrine (noradrenaline) were the most potent effectors of in vitro fluid secretions (Figure 2). These results suggest for the first time that catecholamines are likely the endogenous stimulant for hard tick saliva secretion and thus the salivary gland acts as an active secretory organ. In addition, 5-hydroxytryptamine (serotonin) was effective only in millimolar concentrations, while cAMP or DL-dopa failed to stimulate fluid secretion. Furthermore, the previously identified [16] in vivo stimulant, pilocarpine, failed to trigger salivation in vitro (Figure 2). The discrepancies between the in vivo and in vitro actions of this drug indicated the existence of pilocarpine-activated neurons in the tick central nervous system, the synganglion, stimulating the as-yet unidentified axons reaching the salivary glands. Thus, if these putative salivary gland-innervating axons are disrupted, as is the case in the in vitro assay, cholinomimetics would fail to stimulate salivary secretion [19]. Unfortunately, this interesting feature of hard tick physiology still remains to be clarified (see the section Impact and Follow-Up Research).

In the same study [19], the authors also tested whether hormonal factors could play a role in salivary gland-mediated secretion. Specifically, they predicted that a putative “salivation hormone” may be present in the hemolymph of feeding females that are in an active salivation period. Interestingly, this assertion was not confirmed as the hemolymph from spontaneously salivating females failed to trigger secretion when applied to isolated glands. Although the authors speculated about the possible degradation of the “salivation hormone” in the hemolymph, their observations led to the first suggestion of a neural (rather than hormonal) control of hard tick salivary glands, where the primary neurotransmitter possesses a catecholaminergic nature (see the section Impact and Follow-Up Research).

The last report of the classic trilogy [20] investigated the influence of monovalent ions and osmotic pressure on salivary secretion. First, it was shown that salivary concentrations of chloride, sodium, and potassium are relatively stable over the course of 6-hour-adrenaline-stimulated secretion of isolated glands of partially-fed *D. andersoni*. Then, when monitoring the adrenaline-mediated secretion rate, either chloride or bromide appeared to be an essential substance for fluid secretion, as secretion was dramatically abolished when replaced by nitrate or acetate. In addition, an interesting finding was that secretion rates of both fluids and chloride retain a linear relationship to chloride concentrations in the bathing media. This feature led to the hypothesis that fluid secretion directly depends upon chloride concentration, and is likely maintained via a chloride pump in the glands that consequently facilitates/inhibits fluid movement. Moreover, the stimulatory or inhibitory effect of high or low potassium concentrations (in the presence of sodium) on fluid secretions, respectively, in the bathing media suggested an active role for a sodium-potassium ion pump (Na^+^/K^+^-ATPase) in the hard tick salivary gland secretory processes. This assumption was confirmed by the complete inhibition of fluid secretion when a plant-derived cardiac toxin—ouabain, a known Na^+^/K^+^-ATPase pump inhibitor—was applied (see the section Impact and Follow-Up Research). Furthermore, using a microelectrode to measure the potential differences across the acinar epithelium of the isolated salivary glands demonstrated a doubled (35 mV) potential in the glands actively secreting saliva (induced by adrenalin) compared to the resting, non-secreting glands. Although the authors did not define the specific type of acinus among the three being measured in this study, the potential value differences clearly indicated a relationship with ion secretion.

These series of elegant experiments [20] suggested the passive movement of water from the exterior via the salivary gland epithelium due to the active transport of solute ions. Subsequently, this conclusion raised an important question of how the secreted saliva appears to be hypo-osmotic, rather than hyper/iso-osmotic, compared with the bathing medium. Although not experimentally proven in the study, the authors [20] postulated an inventively intuitive hypothesis where solute ion reabsorption occurs from hyper-osmotic primary saliva that forms in the acinar lumen, somewhere between the secretory acini and the orifice of the main salivary duct (see the section Impact and Follow-Up Research).

## 3. Impact and Follow-Up Research

The studies of Kaufman and Phillips in 1973 [18,19,20] utterly impacted and shaped today’s tick salivary gland physiology research field. As an immediate consequence of these studies, two main research groups began to accelerate advances in this subject area. One was led by Dr. Kaufman himself (University of Alberta, Edmonton, Canada), and the other by Dr. Sauer (Oklahoma State University, Stillwaters, OK, USA). They contributed to the field with multiple relevant reports principally focused on elucidating the control mechanisms of the salivary gland secretions [24,25,26,27,28,29,30,31,32,33,34,35,36,37]. Several other research teams accompanied these achievements up to the early 1990s, with an excellent series of tick salivary gland ultrastructural studies, corroborating the conclusions from both groups [37,38,39,40,41,42] and thus further advancing the cell biology and physiological knowledge of ixodid tick salivary glands.

After the classic foundational trilogy [18,19,20], Kaufman primarily concentrated his scientific focus on discovering the controlling mechanism that triggers salivation, known as catecholaminergic control of hard tick salivary gland fluid secretion [18,32]. In parallel, he was industriously studying the physiology of pilocarpine-induced salivary gland secretion [34], often combining both subjects, along with a variety of other fluid secretion-stimulating or -blocking agents into the same study [33,36,43,44]. Additionally, Kaufman’s investigations allowed for the identification of sensory pathways that can influence the salivary gland fluid secretions [43]. The precise and innovative nature of Kaufman’s experiments, largely employing in vivo and in vitro pharmacology in combination with approaches directly relevant to the investigation of hard tick salivary fluid secretion, resulted in an impressive collection of publications providing invaluable information on hard tick salivary gland physiology [11,25,26,29]. Within these studies, endogenous tick dopamine and the exogenous cholinomimetic drug, pilocarpine, were identified as the most potent stimulants of salivary gland fluid secretion in hard ticks [18,26,33,34,43]. At this point, it is significant to mention that based on these results, multiple laboratories worldwide currently use dopamine or pilocarpine agents to obtain tick saliva for further analyses. Furthermore, Kaufman’s body of work also influenced other groups including Sauer’s to investigate the mechanisms of salivary gland functioning, either on- or off-host, jointly contributing to the general understanding of the vital role played by salivary glands in tick biology [24,25,26,29].

Based on the volume of reports and variety of valuable results obtained between the 1970s and the late 1990s, one could say that the tick salivary gland physiology knowledge had reached a plateau [25,26,29], and that the golden era of tick salivary gland physiology was coming to an end. However, the researchers were limited during this period as they did not have access to today’s highly advanced techniques, so the majority of these studies largely lacked sufficient supporting evidence to form solid conclusions at the molecular, biochemical, or imaging levels. Nevertheless, it is important to highlight that this statement is definitely not intended to demean the scientific merit of this earlier research, which undoubtedly serves as an invaluable stepping stone for further detailed exploration of the fascinating field of tick salivary gland physiology. Here, I briefly discuss a few examples supporting the assertion that earlier investigations have been complemented, clarified, or even revised by recent or current studies.

The publication of Kaufman and Phillips’ 1973 body of work [18] provided concrete evidence that dopamine is likely the primary endogenous stimulant of tick salivary gland fluid secretion [24,32,33]. Subsequently, it has generally been agreed that synaptic dopamine arising from the dopaminergic neurons of the tick central nervous system activates a putative postsynaptic D1 subtype of dopamine receptors, presumably located on the membrane of the salivary gland cell(s) in type II and III acini. Furthermore, the receptor was thought to be coupled to the cAMP pathway, triggering fluid secretion and the Ca^++^ pathway, stimulating the secretion of prostaglandin E2 (PGE2). Consequently, PGE2 stimulates protein secretion in neighboring cell(s) via intracellular calcium mobilization [28]. Interestingly in 1999, Kaufman et al. [45] identified the salivary glands as a major pool of dopamine, but were unable to conclude whether dopamine interferes with “salivary nerves” that were believed to be dopaminergic [26,32]. Access to the *Ixodes* genome sequence [46,47] led to the identification of two different dopamine receptors, D1, acting via the cAMP pathway, and the invertebrate specific D1-like (InvD1L), acting via the Ca^++^ pathway, both stimulated by paracrine/autocrine signals in types II and III salivary gland acini [48,49,50]. In addition, the influx of fluids by type II/III acini and the pumping/gating actions of type III acini occur as an output of the activation and signaling cascade of the epithelial D1 and axonal InvD1L dopamine receptors, respectively [51]. This research clarified the actions of dopamine-mediated hard tick salivary gland secretion and significantly revised the previous model.

In 1973, Kaufman and Phillips [20] suggested a role for a putative Na^+^/K^+^-ATPase pump in ion transfer in adrenaline-stimulated salivary glands. Four decades then passed until the tick Na^+^/K^+^-ATPase was molecularly identified [52] and subsequently localized at the epithelium of all three types of acini in the *Ixodes* female salivary glands. The same study confirmed that in fed ticks, Na^+^/K^+^-ATPase acts downstream of dopamine-mediated salivary secretion, resulting in the formation of a hyperosmotic sodium-rich primary saliva in acinar lumen that are then expelled to the ducts. A completely novel discovery was that type I acini, located exclusively on the proximal part of main salivary duct, use Na^+^/K^+^-ATPase pumps to resorb ions from the primary saliva in the main duct [52]. These studies further support the hypothesis of Kaufman and Phillips (1973) [20] that ion reabsorption occurs somewhere between the secretory acini and duct orifice, and led to a completely new perspective on the role of type I acini in salivary gland physiology. Specifically, older histological investigations hypothesized that in desiccated conditions, type I acini may secrete hygroscopic saliva, forming humidity-binding crystals onto their hypostomal surface during the off-host period, which could then subsequently be swallowed to rehydrate [53,54,55]. However, studies by Kim et al. [52,56,57] provided considerable evidence that during both on- and off-host periods, the production of hyper-osmotic primary saliva is exclusively regulated by type II/III acini. In feeding ticks, ion absorption by type I acini enables the secretion of hypo-osmotic saliva, whereas in desiccated ticks, these structures remain inactive to ensure that hyper-osmotic saliva is passed to the mouthparts to form hygroscopic substances [57].

The inability of pilocarpine to activate salivary gland secretion exclusively in vitro (Figure 2)*,* as observed by Kaufman and Phillips (1973) [19], created another line of unique research in the field of hard tick salivary gland physiology. Based on multiple reports and reviews [26,29,34], it has been suggested that pilocarpine-mediated salivation in vivo is an indirect process, likely linked to an activation of the putative muscarinic-acetylcholine receptor (mAChR) in tick synganglion, which subsequently stimulates an unidentified non-cholinergic “secreto-motor” nerve, directly innervating secretory acini in the salivary glands [26,34]. Kaufman et al. [43] proposed that this cholinergic signal may arrive as an output of upstream sensory system(s) with yet unknown physiological significance. Furthermore, in the same study, they suggested the existence of putative stretch receptors that likely mediate the hemolymph volume in feeding ticks via the activation of the “secreto motor” nerve by means of an unknown transmitter [43]. Unexpectedly, the recent 2020 report by Mateos-Hernandéz et al. [58] described a direct cholinergic connection between tick synganglion and type I acini via distinct neurons, and identified two different types of mAChR expressed in the *Ixodes* salivary glands. This innervation has been associated with water uptake in desiccated ticks, supporting the recent conclusions regarding the role of type I acini [58]. Although these findings do not unequivocally support the proposed model of the indirect cholinergic control of hard tick salivary gland secretion, we must still consider the existence of cholinoceptive axons innervating saliva-producing types II and III acini, as suggested by previous studies [26,29,33,34].

Currently, the only axons known to specifically connect distinct cells in tick synganglion with saliva-producing type II/III acini are neuropeptidergic axons [59,60,61,62,63,64]. The discovery of these axonal processes in 2008 [59] unlocked a completely new research horizon in the understanding of the neural control of tick salivary glands. Detailed reports of peptidergic neuronal cell morphology and the association between their axon terminals and specific cells in type II/III salivary gland acini to activate postsynaptic receptors highlight the complexity of hard tick salivary gland control [65,66]. Moreover, the existence of neuropeptides delivered to salivary glands via axonal projections indicates that chemical neurotransmitters are not the sole controllers of tick salivary gland physiology. Here, it would be interesting to investigate whether some of these axons can serve as “secreto-motor” nerves as predicted by Kaufman [32,33,43] and whether they are sensitive to pilocarpine or other compounds. The current research program at my laboratory in France is placing a strong focus on these aspects, with early results promising to clarify these unanswered queries (publication in preparation). Finally, the remaining task for future experiments would be to explore whether certain protein hormones, along with their receptors, may regulate the activity of tick salivary glands.

In summary, tick salivary gland physiology has been extensively studied over the five past decades by numerous research groups, and was forever transformed by the classic 1973 body of work by Kaufman and Phillips [18,19,20]. Currently, the salivary gland is the most intensively studied tissue among all internal tick organs, with a 23 February 2023 PubMed search for “tick salivary glands” returning a total of 1336 publications. This is largely due to the research community’s impressive drive to identify the factors that either modulate host immune responses and/or facilitate pathogen transmission [67] to enable the development of effective control measures for these medically important pathogen-carrying arthropods. To accelerate this global battle against tick-borne disease, one attractive approach could be to target the vulnerable aspects of tick salivary gland physiology. Effectively disrupting their function may have a fatal effect on ticks and subsequently tick-borne pathogen transmission. If and when we succeed, the credit will undoubtedly be shared among the entire worldwide tick research community that have all contributed to a vastly expanded understanding of tick salivary gland physiology.
